# Environmentally sustainable development and use of artificial intelligence in health care

**DOI:** 10.1111/bioe.13018

**Published:** 2022-03-15

**Authors:** Cristina Richie

**Affiliations:** ^1^ Delft University of Technology—Ethics & Philosophy of Technology Delft The Netherlands

**Keywords:** artificial intelligence, carbon emissions, environmental bioethics, environmental sustainability, health care ethics

## Abstract

Artificial intelligence (AI) can transform health care by delivering medical services to underserved areas, while also filling gaps in health care provider availability. However, AI may also lead to patient harm due to fatal glitches in robotic surgery, bias in diagnosis, or dangerous recommendations. Despite concerns ethicists have identified in the use of AI in health care, the most significant consideration ought not be vulnerabilities in the software, but the environmental impact of AI. Health care emits a significant amount of carbon in many countries. As AI becomes an essential part of health care, ethical reflection must include the potential to negatively impact the environment. As such, this article will first overview the carbon emissions in health care. It will, second, offer five reasons why carbon calculations are insufficient to address sustainability in health care. Third, the article will derive normative concepts from the goals of medicine, the principles of biomedical ethics, and green bioethics—the very locus in which AI in health care sits—to propose health, justice, and resource conservation as criteria for sustainable AI in health care. In the fourth and final part of the article, examples of sustainable and unsustainable development and use of AI in health care will be evaluated through the three‐fold lens of health, justice, and resource conservation. With various ethical approaches to AI in health care, the imperative for environmental sustainability must be underscored, lest carbon emissions continue to increase, harming people and planet alike.

## 1 INTRODUCTION

Artificial intelligence (AI) refers to the way machines and technologies observe, learn, and react. AI systems are developed by human designers, which then are used in a variety of ways, from internet search engines, to robots, to problem‐solving tasks. In 2021, Bélisle‐Pipon et al. argued that AI is exceptional in health technology assessment for five discrete reasons, including ethical challenges.^1^ Indeed, health care and modern medicine can and do avail themselves of AI in a variety of ways, with varying degrees of ease, safety, and ethics. For instance, AI has the capacity to transform health care by delivering medical services to underserved areas, thus improving health outcomes for the poor and vulnerable,^2^ while also filling gaps in health care provider availability, thus better meeting the medical needs of low and middle income countries, as well as aging and rural populations.^3^ However, AI may also lead to patient harm due to fatal glitches in robotic surgery,^4^ bias in diagnosis,^5^ or dangerous recommendations,^6^ among others.

Despite concerns ethicists have identified in the use of AI in health care, the most significant consideration ought not be vulnerabilities in the software like data manipulation, privacy breaches, or potential for exploitation of biodata, but the environmental impact of AI. Health care emits a significant amount of carbon in many countries, but the environmental impact of health care has been underconsidered, in part, because of the assumption that all available health care technologies are medically necessary and therefore carbon emissions are morally irrelevant. As such, when the carbon impact of health care *is* evaluated, it is primarily at the institutional level—that is, the carbon of hospital buildings.^7^ This paradigm circumvents accountability for the environmental impact of health care delivery, even though hospital care and physician and clinical services are the two largest carbon contributors to health care—exceeding health care buildings.^8^

As AI further becomes an integrated and essential part of health care delivery, ethical reflection must include the potential to negatively impact the environment. Carbon emissions from AI use appear throughout the lifecycle of programming, development, and use due to the high energy and resource demands of AI. For instance, 40 days of training Google's AlphaGo Zero game was comparable to the carbon impact of 1,000 hr of air travel.^9^ All AI systems must go through programming, running, and training. Moreover, the extraction of minerals, metals, and plastics necessary for AI capable hardware has tremendous environmental implications.^10^ By way of illustration, Kate Crawford and Vladan Joler traced the environmental impacts of an Amazon Echo, illuminating the vast web of material resources used for extraction and production.^11^ More robust research on the environmental impacts of AI were published by Crawford in 2021.^12^ The carbon impact of health care and biotechnologies is a rapidly developing bank of knowledge in biomedicine, but it is insufficient to address comprehensive sustainability in health care, which requires an ethical framing that goes beyond carbon emissions and is sensible within technological ethics, as well as biomedical and environmental ethics.

Thus, this article will first overview the current output of carbon emissions in health care. It will, second, offer five reasons why carbon calculations are insufficient to address sustainability in health care. Third, the article will derive normative concepts from the goals of medicine, the principles of biomedical ethics, and green bioethics—the very locus in which AI in health care sits—to propose health, justice, and resource conservation as criteria for sustainable AI in health care. In the fourth and final part of the article, examples of sustainable and unsustainable development and use of AI in health care will be evaluated through the three‐fold lens of health, justice, and resource conservation.

There is international agreement that AI should be used ethically, despite competing criteria.^13^ Many of these guidelines include sustainability and justice,^14^ but outside of the ethics of technology other stakeholders, such as the medical industry, may prioritize different criteria. With various ethical approaches to AI, health care, and AI in health care, the imperative for environmental sustainability must be underscored, lest carbon emissions continue to increase, harming people and planet alike.

## 2 HEALTH CARE AND CARBON EMISSIONS

Safe amounts of carbon in the atmosphere have been exceeded, in part, because of medical lifestyles in the industrialized world. The United States health care industry expends an estimated 479 million metric tons (MMT) of carbon dioxide per year; nearly 8% of the country's total emissions. Compare this with China's medical carbon, at 600 MMT, or 6.6% of the country's emissions,^15^ and the United Kingdom's National Health Services (England) medical carbon at 27 MMT.^16^ Carbon dioxide emissions do not stay within national borders. The health impacts of climate change are often linked to environmental racism^17^ and social determinants of health^18^ through the increase in climate‐change related health hazards.^19^ Environmental racism refers to environmental health hazards, such as toxic waste sites and residential areas prone to flooding and mold, that cluster in low‐income areas that tend to have higher populations of racial and ethnic minorities. Social determinants of health like education, income, and vocation—also tied to race and ethnicity—place minorities at risk for certain noncommunicable diseases like obesity and heart disease, while also making positive health care outcomes less likely. Climate‐change related health hazards, such as death and injury from flooding, heat waves, and poor nutritional quality of food because of pests also affect vulnerable populations more dramatically than privileged residents who have the money, resources, or political power to relocate, access better health care, and purchase higher quality food. Thus, the effects of carbon emissions are disproportionately burdensome to the poor nationally and internationally.

A decade ago, very few medical developments, techniques, and procedures had carbon numbers attached to them. Now, it is more common to find data on the environmental impact of health care delivery using the standard metric of carbon dioxide emissions (CO_2_).^20^ Medical developments, techniques, and procedures are calculated on a medical lifecycle from initial doctor's appointment, to procedures, to follow‐up care. For instance, a dental examination emits 5.5 kg of CO_2_.^21^ The carbon impact of a cesarian section is 47.1 kg, nearly triple that of natural vaginal childbirth, which is 17.3 kg of CO_2_.^22^ A cataract operation emits 181.8 kg of CO_2_.^23^ Inpatient admission to a hospital, based on admission intake plus 3.6 bed days per admission, emits 380 kg of CO_2_ per patient.^24^ A heart bypass operation emits 1.1 t of CO_2_.^25^ Conventional hemodialysis for kidney disease emits 10.2 t of CO_2_ per patient annually.^26^ Notably, while all medical procedures have a carbon footprint, only assisted reproductive technologies (ARTs) have a carbon legacy—the lasting impacts of carbon choices through the addition of a person, who will likely reproduce as well. The carbon footprint of each ART infant in the United States is 1,644 t of CO_2_ per child, with a carbon legacy of 9,441 metric tons of carbon dioxide for the adult who uses ARTs, based on average reproduction rates.^27^

Studies have been done on the carbon impact of AI in health care, with particular focus on telemedicine. In 2012, Holmner et al. recorded that the carbon cost of 238 telemedicine appointments in Sweden was 602 kg CO_2_ with a range of 1.86–8.43 kg CO_2_ for a 1‐hr telemedicine appointment.^28^ Later, in a comprehensive comparison study, Purohit et al. found that the carbon footprint savings from using telemedicine range between 0.70 and 372 kg CO_2_ per consultation.^29^

### 2.1 Carbon as an inadequate criterion for sustainability

In 2021, Tsagkaris et al. argued that AI can reduce the carbon of health care.^30^ And, while carbon emissions are one of the most widely accepted, and in some aspects, simplest metric to assess the sustainability of a particular good or service, using CO_2_ as a criterion for health care sustainability is impractical—and ethically insufficient—for several reasons, despite it remaining the standard measurement in academic discourse.

First, although the carbon emissions of some health care developments, techniques, and procedures have been calculated, the vast majority have not. Calculating carbon numbers on all aspects of health care, in every country, and each branch of medicine will take an enormous amount of time and human resources. Like all carbon calculations, numbers are highly variable based on location (i.e., carbon of the country, national environmental standards) and available data. Moreover, carbon calculations can be elusive: just as hard data appears the inputs change. For instance, a carbon calculation of a cataract surgery will vary based on available resources, human efficiency, patient medical condition, and sourcing of energy. Despite this gap in knowledge, humankind must not wait for empirical data on carbon before making changes in consumption habits. As a society we know we must reduce our carbon. Climate change is too urgent a matter to wait for carbon calculations to justify sustainable health care.

Second, the motivation for carbon calculations is to reduce carbon either through carbon capping or carbon allocation. However, this assumes that there is a sustainable amount of carbon that can be emitted on a yearly basis. This is untrue. The amount of “safe” carbon in the atmosphere—calculated to be 350 parts per million—has already been exceeded.^31^ Allocating carbon to each country does not work within the current environmental problem that requires a zero, or negative, emission solution. More significantly, however, is that no amount of voluntary carbon capping will affect sustainability unless the major polluters in the world—the United States and China—reduce their emissions.

Third, and related to the second point, there is a concern that carbon calculations will lead to unfair limitation in health care.^32^ Indeed, bias and discrimination that would lead policymakers to deprioritize health care needs of the medically underserved, including women,^33^ LGBTQ+,^34^ the disabled,^35^ and racial minorities,^36^ must be avoided. However, health care carbon expenditure does not necessarily translate to better medical quality of life. In the United States, disparities between substantial health care emissions and poor health outcomes point to health care waste and misdistribution of medical resources. Increasing the carbon emissions of health care will do little to support positive health care outcomes if social determinants of health and bias are unaddressed.^37^^,^ 38 ^,^ 39 ^,^ 40 Carbon emissions alone are inadequate in capturing other ethically significant factors, such as distributive justice and competing moral values.

Fourth, while carbon is often tied to environmental ethics, carbon calculations are morally reductionistic and fail to inculcate virtue into a person. Reducing environmental ethics to the carbon number associated with a given item absolves individuals from thoughtful consideration of consumption habits. While the outcome might be a more sustainable planet, society should not have to sacrifice moral development in the pursuit of ecology. Inner motivation for conservation will outlast the immediate environmental problem, which can in turn prevent another environmental crisis. Carbon calculations are disposable in an ethical system that does not find intrinsic value in a clean, healthy planet. Self‐directed sustainability addresses the underlying problem by changing values and therefore habits and actions.

Fifth, while carbon as a metric does provide information on the environmental impact, simply identifying a carbon number and then declaring an item “sustainable” or “not” is meaningless since we are beyond the point of finding a carbon equilibrium and must live in a carbon recession. A carbon number, much like caloric information on food packaging, is merely descriptive unless it is set within a normative context. Unlike calories, however, there is no recommended daily carbon emissions that can be sustainably produced. Since carbon numbers are a limited, at best, way to determine sustainability, another approach must be taken.

## 3 HEALTH, JUSTICE, AND RESOURCE CONSERVATION AS CRITERIA FOR SUSTAINABILITY

In health care, the goals of medicine^41^ are one of the guiding frameworks for the delivery and development of medicine and associated technologies. The goals of medicine are supported by the principles of biomedical ethics, which are broadly embraced as having normative significance.^42^ Both will be discussed more in the following sections, recognizing that they are incomplete paradigms for sustainable health care since the environmental impact of health care was not considered in the development of either. In response to this oversight, environmental bioethics, which examines the health impacts of climate change, developed.^43^ Later, green bioethics and other paradigms inverted the environmental concern from health impacts of climate change to the environmental impact of health care, or how health care contributes to climate change through resource use.^44^

Instead of compartmentalizing these frameworks, the goals of medicine, the four principles of biomedical ethics, and green bioethics taken in aggregate represent standards in health care delivery, biomedical ethics, and environmental bioethics, respectively. Moreover, they are philosophically sound and invoke larger ethical values, making them commensurate with each other. Within these three frameworks, there are certain ideas that dominate; an essence that underpins the commitments of each. Arguably, the goals of medicine, the four principles of biomedical ethics, and green bioethics can be summarized by a commitment to health, justice, and resource conservation, respectively. These key terms, understood within their broader context, can guide the ethical assessment of sustainable development and use of AI in health care without relying on carbon calculations, which as argued earlier, have significant limitations. Since the terms are broad, conflicts of values should be minimized. In places of ostensible conflict, for instance, health of the individual or resource conservation, it should be remembered that the health of individuals is very often dependent on resource conservation and thus both can be reconciled.

### 3.1 The goals of medicine: Health

The traditional “goals of medicine,” identified by Joseph H. Howell and William Frederick Sale, provide the scope for health care delivery. The goals of medicine are

the prevention of disease and injury and the promotion and maintenance of health; the relief of pain and suffering caused by maladies; the cure of those with a malady, and the care of those who cannot be cured; and the avoidance of premature death and the pursuit of a peaceful death.^45^

These goals can be summarized by the word “health,” which endures as the reference point for medicine.

Although the term “health” can be highly individualistic and contingent on available resources, social location, age, sex, ability, and personal desires,^46^ health nevertheless joins together the pursuit of medicine with the delivery of health care. Health is regarded as a significant component of human life, with numerous medical organizations dedicated to human health worldwide, such as the World Health Organization, Médecins Sans Frontières, and the Joint United Nations Programme on AIDS. Health as a broad term does not specify how health should be maintained or obtained, thus it is also wide enough to encompass the scope of health care and the pursuits thereof.

### 3.2 Principles of biomedical ethics: Justice

The pursuit of health as a goal of medicine enjoys great consensus. In pursuit of this goal, standards of ethical guidance have been developed locally and globally from diverse streams of thought.^47^ Modern, Western health care has generally derived ethical guidance from the four principles of biomedical ethics—respect for autonomy, beneficence, non‐maleficence, and justice—proposed by Tom Beauchamp and James Childress at Georgetown University.^48^ These principles were thought to express prima facie morality, though the geographical, historical context in which they emerged cannot be ignored. Respect for autonomy is often emphasized in applications of biomedical ethics.^49^ However, justice is a better representative of biomedical ethics, since it thematically encompasses the other principles.

### 3.3 Green bioethics: Resource conservation

The convergence of medicine, health care ethics, and environmental ethics was originally part of both Fritz Jahr^50^ and Van Rensselaer Potter's work on bioethics,^51^ the latter of which drew on conservationist Aldo Leopold's environmental land ethic.^52^ Jahr and Potter's work later developed into global bioethics, then became relegated to environmental bioethics as Beauchamp and Childress' principles of biomedical ethics dominated in academic and clinical practice of ethics. The current environmental crisis has largely been ignored by traditional biomedical ethics,^53^ hence, green bioethics emerged from the pressing need for a coherent ethical framework for sustainability in health care.^54^ Drawing on the wisdom of environmental ethics and the scope of biomedicine, green bioethics—developed by Cristina Richie—has offered four principles for assessing the environmental sustainability of medical developments, techniques, and procedures.

The first principle of green bioethics—distributive justice—locates biomedical obligations within a global society. Distributive justice entails mitigating gaping disparities in health care delivery. This occurs when basic medical developments, techniques, and procedures are allocated to all people before the financial elite utilize medical developments, techniques, and procedures that do not cure, treat, or prevent diseases.

The second principle of green bioethics—resource conservation—states that human health care needs should be given priority before human health care wants. Expansion of health care needs will not conflict with environmental conservation if health care wants are limited.

The third principle of green bioethics—simplicity—occurs through the prevention of diseases and a gradational approach to medical interventions. If prevention of disease is not possible, the principle of simplicity, or therapeutic parsimony, works through less resource intensive, less invasive, or less complex options before escalating to resource intensive, invasive, or complex interventions.^55^ Simplicity undercuts the current model of high‐tech, maximalistic health care delivery, which causes unnecessary resource consumption.

The fourth principle for green bioethics—ethical economics—argues that humanism should drive health care developments before profitability. Financial gain often determines which medical developments, techniques, and procedures proliferate and which remain dormant. As a result, elective procedures that do not cure, treat, or prevent disease are readily available for those who can pay while the increasing cost of life‐saving medicine prevents the poor from receiving health care. Ethical economics is not opposed to generating revenue, but health care must not lose its primary mission of health and healing.

If applied correctly, resource conservation is the outcome of the four principles of green bioethics and the measure of their efficacy. Thus, it is the most appropriate representative principle.

## 4 SUSTAINABLE AND UNSUSTAINABLE DEVELOPMENT AND USE OF AI IN HEALTH CARE

The lexicon of the goals of medicine, biomedical ethics, and green bioethics capitalizes on foundational efforts to codify normative commitments. Health, justice, and resource conservation have been highlighted as thematic guides in this article, which map on to each of the three ethical systems named above. Health as a criterion for the sustainable development and use of AI does not necessarily place sickness as an antonym, although it may be the case that AI causes medical error. Rather, development or use beyond the purposes of health—whether enhancement, pleasure, or luxury—ought to be regarded as the unsustainable twin of health. Justice is a necessary component of non‐individualistic sustainable artificial intelligence in health care, since it recognizes the claims of others. Biomedical ethics cannot pursue that which is unjust, or contributes to injustices. Thus, AI that widens health care gaps may be unjust and unsustainable. Resource conservation might occur in various steps along the health care delivery chain. AI could make a technique or a medical process more sustainable by conserving raw materials or reducing the need for medical interventions.

There are a number of possible weaknesses with this ethical framework at the conceptual level. The criterion of health is by no means a broadly agreed upon term, despite the contextualization given above.^56^ Moreover, health encompasses many physical, emotional, spiritual, and psychological aspects.^57^ The ordering of these aspects in proximity to the goals of medicine is debatable. Thus, even if different aspects of health were accounted for in AI, the ranking that an AI program might give to a particular aspect of health may be incongruent with patient preferences.^58^ AI ordering, even if it accounted for a “standard patient” (which is a very nebulous concept, indeed) may be irrelevant if the health care facility does not have the means to support that particular aspect of health, or if the health care providers do not have the competencies in that particular area.^59^ For example, an AI algorithm that has determined a tracheostomy will eventually restore health may be against a patient's do not intubate order, or unavailable in that facility, or not part of the health care provider's skill set.

These conceptual problems of health are applicable to the terms justice and sustainability as well. Justice is particularly elusive as a concept^60^ and sustainability a less recognized ethical value in health care.^61^ Although there are numerous reasons to include sustainability as an essential criterion for AI ethics, enumerated above, many health care systems are reluctant to include it when evaluating the ethics of medical decisions. One notable exception is the United Kingdom's National Health Service (NHS), which adheres to legally binding carbon reduction measures,^62^ indicating that sustainable health care is a cornerstone of modern medicine.

For the purposes of this application, “health care” will include: (a) health care systems, such as hospitals and clinics; (b) medical procedures used within health care systems, such as robotic surgery and implantation of monitoring devices; and (c) health care insurance organizations, such as Blue Cross Blue Shield and the NHS, that collect and store health data.

To be sure, this excludes a number of places where health care is delivered in less formal settings, for instance home care and holistic doctors' offices.^63^ This also excludes health care delivery outside of a medical setting, such as emergency medical services, which may nonetheless have access to AI.^64^ Furthermore, many forms of technology collect and store health data—from Apple watches to Google searches for medical questions.^65^ However, systems, procedures, and insurance define the parameters of health care in many countries where AI is being developed and used, thus providing a first place for inquiry, based on the potential for mass utilization of AI biotechnologies and environmental impact.

“Sustainable development” refers to theoretical AI. These are forms of AI that are being considered for development or expansion. “Sustainable use” refers to applied AI. These are the ways that AI—once ready for deployment—is actually used in health care. The line here is discreet. The time between development and use is not fixed, as there is a period of trial and error in any new technology.^66^ Moreover, differing rates of development across interdependent sectors in health care biotech, for example bioengineering and computational mathematics, place development and use of AI as an ever moving field of ethical inquiry.^67^ There are also differences of opinion among stakeholders about which aspects of AI should be developed, which will set the tone for health care innovation.^68^ These complications are compounded given the various objectives that global partnerships and country‐specific research teams must meet in AI development.^69^ AI technologies are rapidly progressing. As they advance, a measure of agility in ethical application is required.

### 4.1 Sustainable development of AI in health care

Sustainable development of AI in health care may include, for instance, triage algorithms in emergency rooms.^70^ Development of triage algorithms could produce a template for appropriate medical care, which would support the goals of health. The algorithm could facilitate equity in wait times, which would uphold the biomedical principle of justice. Triage algorithms in emergency rooms could be calibrated for allocation of resources with the highest clinical impact, thus conserving resources for high‐success procedures.

At the same time, algorithms are programmed by humans and humans are fallible.^71^ Errors might occur if an AI programmer fails to adjust for regional or state‐wide differences in the type of emergency room cases that are frequent in a locale (e.g., frostbite or heatstroke).^72^ The effects of bias, particularly unconscious bias—of sexism, racism, heterosexism, and so forth—may influence the programming people develop.^73^ That is, a person may not be aware of their own biases and develop programs that replicate their own insensitivities. In cases of “deep learning” where AI gathers information from itself and adjusts accordingly, biased inputs at the beginning of the algorithm can contaminate the entire sequence, which can result in biased outputs.^74^

Even in a perfectly designed AI algorithm, efficacy—in terms of health, justice, and sustainability—depends on human execution. This is both a benefit and a burden. On the one hand, health care practitioners retain moral responsibility for how they interpret and apply the results of AI algorithms.^75^ On the other hand, inefficient or deviant implementation of AI algorithms may cause more harm, either through medical error, exacerbating inequality, or producing excess medical waste.^76^ Thus, sustainable development and sustainable use of AI in health care often go hand‐in‐hand.

### 4.2 Sustainable use of AI in health care

Sustainable use of AI in health care may include, for instance, analyzing rich text data to detect emerging outbreaks with novel symptom patterns^77^ or identifying patterns of infection.^78^ This use of predictive analytics can prevent outbreaks, which would support the health of populations. Such use of existing AI may harness data on patterns of infection to deliver rapid treatments, which would ensure biomedical justice for patients expecting timely care. Data on symptom patterns may identify undetected disease and avoid the carbon impact of medical intervention related to late detection, thus supporting resource conservation.

Of course, identification of patterns of infection may not be effective predictors of actual infection. As rich data text relies on tracking people who are already infected—in this example—it is reactive rather than proactive. Both false positives and false negatives may compromise reliability.^79^ Delivery of rapid treatment for the infected person presupposes that care facilities are nearby and stocked with the necessary forms of medical treatment, as well as having trained workers to deliver care. Even when these structures are in place, and rich text data can identify a nearby clinic with appropriate care, it does not follow that a sick person could access that clinic, either for mobility or financial reasons.^80^

Finally, there is a logical tension between claiming AI as a tool for conservation vis‐à‐vis early treatment or prevention^81^ and the recognition that each averted death represents an extended medical carbon footprint. That is, using AI for early treatment may use less resources when compared with late‐but‐successful treatment, but successful treatments result in longer life and therefore more years of health care access and health care resource use.^82^ The conclusion that lifesaving health care should not be provided because humans are environmental liabilities is unacceptable;^83^ prevention and early treatment may cause more resource use in the course of a person's life, but it would be unethical and contrary to the goals of medicine to refuse to treat on the basis of resource use alone. Certainly, high‐impact lifesaving medical treatments could be declined based on a lack of clinical indication or on the basis of futility.^84^ Elective, resource intensive health care may be postponed due to prioritization.^85^ The tension between resource use of medical care and prolonged lifespans does not indicate that using AI for early detection is in‐se unsustainable, but rather a critical reflection of global resource use must be undertaken. Humankind must choose where to use resources; medical care would be less of an environmental concern if other areas of life were sustainable.

### 4.3 Unsustainable development of AI in health care

Contrarywise, unsustainable development of AI in health care may include gene‐editing for aesthetic characteristics.^86^ Superficial characteristics, by definition, fail to address health. This cosmetic option could quickly become a commercialized service that exacerbates inequality through inhibited access, thus ignoring justice. Gene‐editing for aesthetic characteristics has the potential to be used primarily in high carbon countries, hence resources will be exploited rather than conserved.

There are many debates about the value of utilizing “aesthetics” as part of health care.^87^ These arguments are generally put forth in locales where basic health care is available and personal desires drive the pursuit of cosmetic “medicine.”^88^ Another serious concern about limiting unsustainable AI developments is that of moral luck and moral responsibility.^89^ Moral luck is the theory that recognizes that people are born into better or worse circumstances through no merit of their own and moral responsibility therefore attempts to balance these inequities. While those in the developed world who are accustomed to capitalistic choice in a market economy may opine that it is unfair to restrict access to elective treatments on the basis of resource use, this objection is minimized with the recognition that climate change harms everyone. Therefore, so‐called “entitlements” of individuals to particular elective procedures must be balanced with the environmental effects that they—and others—will experience, while also raising the medical standard in places that are underserved.

In many ways, ethical concerns about developing AI for aesthetic characteristics cut across the goals of medicine, biomedical ethics, and green bioethics. The discussion on therapy and enhancement,^90^ access and distribution of medical resources,^91^ and luxury and subsistence emissions,^92^ respectively parallels other ethical concerns about aesthetic medicine. These arguments are reified with each new technological development and deserve reevaluations so long as ethical differences linger. Historical conversations are still relevant, but with additional moral valence assigned to the environmental crisis.

### 4.4 Unsustainable use of AI in health care

Unsustainable use of AI in health care might include Care Bots for children^93^ and other patient populations. Care Bots, which are robots that provide assistance (such as moving an overweight person),^94^ care (such as nursing assistance), or companionship (in the form of an animal or humanoid, for example),^95^ may, through the delivery of under‐supervised medicine, lead to operational errors that could result in damaged health or death. This can lead to more resource use to correct injuries (of course, less resources are used if a person dies, but this cannot be regarded as an ethical, intended, or desirable outcome). Care Bots privilege the financially secure who have access to high‐tech health care facilities, thus increasing pressure on the over‐carbonated medical system and disregarding biomedical justice. And, if susceptible to technological obsolescence, Care Bots may require enormous amounts of resources for updates and replacement.

Using AI in Care Bots may cause medical harm, but like other forms of high‐tech health care, this is a risk that patients might be willing to undertake once properly consented.^96^ The issue of moral luck might also be invoked in support of AI in Care Bots, that if available, they should be used, with the caveat that this form of AI appears to be more “value added,” rather than at the core of medicine, and therefore more subjected to ethical scrutiny.

There are many different types of Care Bots and it may not be the case that all Care Bots would be unsustainable. However, while some technologies are intentionally programmed to be obsolete, all will become obsolete eventually,^97^ causing unnecessary resource use. Whereas human caretakers may need to learn new skills through continuing education, this is a low‐impact activity; re‐programing or updating Care Bots is a more resource intensive endeavor.^98^ A similar but related objection to the unsustainability of Care Bots is the availability of satisfying alternatives. Even with health care provider shortages in some countries,^99^ mobility and training can supply human health care workers to those in need, thus making this form of AI in some ways redundant. Unlike genetic editing, or rapid algorithms, which have less appealing alternatives due to cost, time, or intellectual investment, the functions of many Care Bots can be performed equivalently, if not better, by humans.^100^

These examples of sustainable and unsustainable development and use of AI are entry points for ethical assessment, but far from comprehensive. In the future, the most innovative and ethically complex forms of AI will need to be evaluated based on the criteria of health, justice, and resource conservation.

## 5 CONCLUSION

Consensus about “the” best, or most relevant, ethical system for health care is an ongoing conversation. As AI becomes indispensably ubiquitous, there is societal reticence to discontinue it unless there is a compelling reason. The relationship between technology and ecology will be a defining feature of biomedicine in the 21st century. As such, ethicists are better situated to advocate for the sustainable *use* of established AI rather than persuade society to abandon new developments in AI altogether. By utilizing the criteria of health, justice, and resource conservation the goals of medicine are ethically supported while the possibility—indeed, the necessity—of integrating just sustainability into health care is actualized.

## CONFLICT OF INTEREST

The author declares no conflict of interest.

## Footnotes

1 Bélisle‐Pipon, J.‐C., Couture, V., Roy, M.‐C., Ganache, I., Goetghebeur, M., & Cohen, I. G. (2021). What makes artificial intelligence exceptional in health technology assessment? *Frontiers in Artificial Intelligence, 4*, 736697.

2 Ilan, Y. (2021). Improving global healthcare and reducing costs using second‐generation artificial intelligence‐based digital pills: A market disruptor. *International Journal of Environmental Research and Public Health, 18*(2), 811.

3 Vrontis, D., Christofi, M., Pereira, V., Tarba, S., Makrides, A., & Trichina, E. (2022). Artificial intelligence, robotics, advanced technologies and human resource management: A systematic review. *The International Journal of Human Resource Management*, *33*(6), 1237–1266.

4 Jiang, L., Wu, Z., Xu, X., Zhan, Y., Jin, X., Wang, L., & Qiu, Y. (2021). Opportunities and challenges of artificial intelligence in the medical field: Current application, emerging problems, and problem‐solving strategies. *Journal of International Medical Research, 49*(3), 03000605211000157. https://doi.org/10.1177/03000605211000157

5 Obermeyer, Z., & Topol, E. J. (2021). Artificial intelligence, bias, and patients' perspectives. *The Lancet, 397*(10289), 2038.

6 Bates, D. W., Levine, D., Syrowatka, A., Kuznetsova, M., Craig, K. J. T., Rui, A., Jackson, G. P., & Rhee, K. (2021). The potential of artificial intelligence to improve patient safety: A scoping review. *NPJ Digital Medicine, 4*(1), 1–8.

7 NHS Sustainable Development Unit. (2009). *Saving carbon, improving health: NHS carbon reduction strategy for England*. NHS Sustainable Development Unit.

8 Eckelman, M. J., & Sherman, J. (2016). Environmental impacts of the U.S. health care system and effects on public health. *PLoS One, 1*(6), e0157014.

9 Preetipadma. (2020, April 29). *New MIT neural network architecture may reduce carbon footprint by AI*. Analytics Insight. https://www.analyticsinsight.net/new-mit-neural-network-architecture-may-reduce-carbon-footprint-ai/

10 Farjana, S. H., Huda, N., & Parvez Mahmud, M. A. (2019). Life cycle assessment of cobalt extraction process. *Journal of Sustainable Mining, 18*, 150–161.

11 Crawford, K., & Joler, V. (2018). *Anatomy of an AI system*. https://anatomyof.ai/

12 Crawford, K. (2021). *The atlas of AI*. Yale University Press.

13 Jobin, A., Ienca, M., & Vayena, E. (2019). The global landscape of AI ethics guidelines. *Nature Machine Intelligence, 1*(9), 389–399.

14 Stahl, B. C. (2021). *Artificial intelligence for a better future: An ecosystem perspective on the ethics of AI and emerging digital technologies*. Springer Nature.

15 Pichler, P.‐P., Jaccard, I. S., Weisz, U., & Weisz, H. (2019). International comparison of health care carbon footprints. *Environmental Research Letters, 14*(6), 064004.

16 Sustainable Development Unit for NHS England and Public Health England. (2018). *Reducing the use of natural resources in health and social care: 2018 report*. https://www.sduhealth.org.uk/documents/Policy%20and%20strategy/20180912_Health_and_Social_Care_NRF_web.pdf

17 Zimring, C. (2017). *Clean and white: A history of environmental racism in the United States*. NYU Press.

18 Paavola, J. (2017). Health impacts of climate change and health and social inequalities in the UK. *Environmental Health, 16*, (1), 113.

19 Costello, A., Abbas, M., Allen, A., Ball, S., Bellamy, R., Friel, S., Groce, N., Johnson, A., Kett, M., Lee, M., Levy, C., Maslin, M., McCoy, D., McGuire, B., Montgomery, H., Napier, D., Pagel, C., Patel, J., de Oliveira, J. A. P., … Patterson, C. (2009). Managing the health effects of climate change. *Lancet, 373*(9676), 1693–1733.

20 Shakow, D. (2017). *Climate change and environmental ethics*. Routledge.

21 Duane, B., Lee, M. B., White, S., Stancliffe, R., & Steinbach, L. (2017). An estimated carbon footprint of NHS primary dental care within England. How can dentistry be more environmentally sustainable? *British Dental Journal, 223*(8), 589–593.

22 Campion, N., Thiel, C. L., DeBlois, J., Woods, N. C., Landis, A. E., & Bilec, M. M. (2012). Life cycle assessment perspectives on delivering an infant in the US. *Science of the Total Environment, 425*, 191–198.

23 Morris, D. S., Wright, T., Somner, J. E. A., & Connor, A. (2013). The carbon footprint of cataract surgery. *Eye, 27*(4), 495–501.

24 Tennison, I., & NHS England. (2010, April 9). *Indicative carbon emissions per unit of healthcare activity* (Briefing No. 23). Eastern Region Public Health Observatory_._

25 Berners‐Lee, M. (2010). *How bad are bananas? The carbon footprint of everything*. Profile Book, pp. 131–132.

26 Lim, A. E., Perkins, A., & Agar, J. W. (2013). The carbon footprint of an Australian satellite haemodialysis unit. *Australian Health Review, 37*, 369–374.

27 Murtaugh, P., & Schla, M. (2009). Reproduction and the carbon legacies of individuals. *Global Environmental Change, 19*(1), 14–20.

28 Holmner, Å., Ebi, K. L., Lazuardi, L., & Nilsson, M. (2014). Carbon footprint of telemedicine solutions ‐ Unexplored opportunity for reducing carbon emissions in the health sector. *PLoS One, 9*(9), e105040.

29 Purohit, A., Smith, J., & Hibble, A. (2021). Does telemedicine reduce the carbon footprint of healthcare? A systematic review. *Future Healthcare Journal, 8*(1), e85.

30 Tsagkaris, C., Hoian, A. V., Ahmad, S., Essar, M. Y., Campbell, L. W., Grobusch, L., Angelopoulos, T., & Kalaitzidis, K. (2021). Using telemedicine for a lower carbon footprint in healthcare: A twofold tale of healing. *The Journal of Climate Change and Health, 1*, 100006.

31 Hansen, J., Sato, M., Kharecha, P., Beerling, D., Berner, R., Masson‐Delmotte, V., Pagani, M., Raymo, M., Royer, D. L., & Zachos, J. C. (2008). Target atmospheric CO_2_: Where should humanity aim? *Open Atmospheric Science Journal, 2*, 217–231; U.S. Department of Commerce National Oceanic and Atmospheric Administration Earth System Research Laboratory, Global Monitoring Division. (2019). *Trends in atmospheric carbon dioxide: Recent monthly average Mauna Loa CO*_*2*_. https://www.esrl.noaa.gov

32 Di Paola, M., & Garasic, M. D. (2013). The dark side of sustainability: Avoiding and shortening lives in the Anthropocene. *Rivista Di Studi Sulla Sostenibilita, 3*(2), 59–81.

33 Thiel, C., Eckelman, M., Guido, R., Huddleston, M., Landis, A. E., Sherman, J., Copley‐Woods, N., & Bilec, M. M. (2015). Environmental impacts of surgical procedures: Life cycle assessment of hysterectomy in the United States. *Environmental Science & Technology, 49*(3), 1779–1786.

34 Richie, C. (2016). Lessons from queer bioethics: A response to Timothy F. Murphy. *Bioethics, 30*(5), 365–371.

35 Hall, K. (2014). No failure: Climate change, radical hope, and queer crip feminist eco‐future. *Radical Philosophy Review, 17*(1), 203–225.

36 Barr, D. (2019). *Health disparities in the United States: Social class, race, ethnicity, and the social determinants of health*. Johns Hopkins University Press.

37 Starfield, B. (2000). Is US health really the best in the world? *JAMA, 284*(4), 483–485.

38 Berwick, D. M., & Hackbarth, A. D. (2012). Eliminating waste in US health care. *JAMA, 307*(14), 1513–1516.

39 Dickman, S. L., Himmelstein, D. U., & Woolhandler, S. (2017). Inequality and the health‐care system in the USA. *The Lancet*, *389*(10077), 1431–1441.

40 Apergis, N., Gupta, R., Lau, C. K. M., & Mukherjee, Z. (2018). US state‐level carbon dioxide emissions: Does it affect health care expenditure? *Renewable and Sustainable Energy Reviews, 91*, 521–530.

41 Howell, J. H., & Sale, W. F. (2000). Specifying the goals of medicine. In L. Frith (Ed.), *Life choices*: *A Hastings Center introduction to bioethics* (2nd ed., pp. 62–73). Georgetown University Press, p. 62.

42 Beauchamp, T., & Childress, J. (1979). *Principles of biomedical ethics* (1st ed.). Oxford University Press.

43 Potter, V. R. (1988). *Global bioethics: Building on the Leopold legacy*. Michigan State University Press, p. 2.

44 Richie, C. (2019). *Principles of green bioethics: Sustainability in health care*. Michigan State University Press.

45 Howell & Sale, op. cit. note 41, p. 62.

46 Preamble to the Constitution of the World Health Organization as adopted by the International Health Conference, New York, June 19–22, 1946; signed on July 22, 1946 by the representatives of 61 States (Official Records of the World Health Organization, no. 2, p. 100) and entered into force on April 7, 1948.

47 Sugarman, J., & Sulmasy, D. P. (2001). *Methods in medical ethics*. Georgetown University Press.

48 Beauchamp & Childress, op. cit. note 42.

49 Holm, S. (1995). Not just autonomy‐The principles of American biomedical ethics. *Journal of Medical Ethics, 21*(6), 332–338.

50 Jahr, F., & Sass, H.‐M. (2010). Bio‐ethics—Reviewing the ethical relations of humans towards animals and plants. *JAHR‐European Journal of Bioethics, 1*(2), 227–231. Originally published as Jahr, F. (1927). Bio+Ethik. Eine Umschau uber die ethischen Beziehungen des Menschen zu Tier und Pflanze, *Kosmos. Handweise fur Naturfreunde und Zentralblatt fur das naturwissenschaftliche Bildungs‐ und Sammelwesen Kosmos: Gesellschaft der Naturfreunde*, 2–4.

51 Potter, V. R. (1982). Bioethics: The science of survival. *Perspectives in Biology and Medicine, 14*(1), 127–153.

52 Potter, V. R. (1999). Fragmented ethics and bridge bioethics. *Hastings Center Report, 29*(1), 38–40.

53 Reich, W. T. (1995). The word ‘bioethics’: The struggle over its earliest meanings. *Kennedy Institute of Ethics Journal, 5*(1), 19–34.

54 Richie, op. cit. note 44.

55 Kelly, J. (2021). The diagnostic approach in complex patients: Parsimony or plenitude? *The American Journal of Medicine, 134*(1), 11–12.

56 Huber, M., Knottnerus, J. A., Green, L., van der Horst, H., Jadad, A. R., Kromhout, D., Leonard, B., Lorig, K., Loureiro, M. I., van der Meer, J. W. M., Schnabel, P., Smith, R., van Weel, C., & Smid, H. (2011). How should we define health? *BMJ, 343*, d4163.

57 Callahan, D. (1973). The WHO definition of ‘health’. *Hastings Center Studies, 1*(3), 77–87.

58 Jha, S., & Topol, E. J. (2018). Information and artificial intelligence. *Journal of the American College of Radiology, 15*(3), 509–511.

59 Guo, J., & Li, B. (2018). The application of medical artificial intelligence technology in rural areas of developing countries. *Health Equity, 2*(1), 174–181.

60 De Schutter, H. (2007). Language policy and political philosophy: On the emerging linguistic justice debate. *Language Problems and Language Planning, 31*(1), 1–23.

61 Bonevac, D. (2010). Is sustainability sustainable? *Academic Questions, 23*(1), 84–101.

62 NHS Sustainable Development Unit. (2009). *Saving carbon, improving health: NHS carbon reduction strategy for England*. NHS Sustainable Development Unit.

63 Shim, J.‐M., & Kim, J. (2018). Cross‐national differences in the holistic use of traditional East Asian medicine in East Asia. *Health Promotion International, 33*(3), 536–544.

64 Grekousis, G., & Liu, Y. (2019). Where will the next emergency event occur? Predicting ambulance demand in emergency medical services using artificial intelligence. *Computers, Environment and Urban Systems, 76*, 110–122.

65 Sharon, T. (2016). The Googlization of health research: From disruptive innovation to disruptive ethics. *Personalized Medicine, 13*(6), 563–574.

66 Tang, X., Li, X., Ding, Y., Song, M., & Bu, Y. (2020). The pace of artificial intelligence innovations: Speed, talent, and trial‐and‐error. *Journal of Informetrics, 14*(4), 101094.

67 Mittelstadt, B. D., Allo, P., Taddeo, M., Wachter, S., & Floridi, L. (2016). The ethics of algorithms: Mapping the debate. *Big Data & Society, 3*(2), 2053951716679679. https://doi.org/10.1177/2053951716679679

68 Preece, A., Harborne, D., Braines, D., Tomsett, R., & Chakraborty, S. (2018). Stakeholders in explainable AI. *arXiv* preprint arXiv:1810.00184. https://arxiv.org/abs/1810.00184

69 Cath, C., Wachter, S., Mittelstadt, B., Taddeo, M., & Floridi, L. (2018). Artificial intelligence and the ‘Good Society’: The US, EU, and UK approach. *Science and Engineering Ethics, 24*(2), 505–528.

70 Bates, D. W., Saria, S., Ohno‐Machado, L., Shah, A., & Escobar, G. (2014). Big Data in health care: Using analytics to identify and manage high‐risk and high‐cost patients. *Health Affairs, 33*(7), 1123–1131.

71 Osoba, O. A., & Welser IV, W. (2017). *An intelligence in our image: The risks of bias and errors in artificial intelligence*. Rand Corporation.

72 Yu, K.‐H., Beam, A. L., & Kohane, I. S. (2018). Artificial intelligence in healthcare. *Nature Biomedical Engineering, 2*(10), 719–731.

73 Simonite, T. (2018, January 11). When it comes to gorillas, Google photos remains blind. *Wired*. https://www.wired.com/story/when-it-comes-to-gorillas-google-photos-remains-blind/

74 Dastin, J. (2018, October 11). *Amazon scraps secret AI recruiting tool that showed bias against women*. Reuters. https://www.reuters.com/article/us-amazon-com-jobs-automation-insight-idUSKCN1MK08G

75 Shortliffe, E. H., & Sepúlveda, M. J. (2018). Clinical decision support in the era of artificial intelligence. *JAMA, 320*(21), 2199–2200.

76 Campion, N., Thiel, C. L., Woods, N. C., Swanzy, L., Landis, A. L., & Bilec, M. M. (2015). Sustainable healthcare and environmental life‐cycle impacts of disposable supplies: A focus on disposable custom packs. *Journal of Cleaner Production, 94*, 46–55.

77 Maojo, V., & Martin‐Sanchez, F. (2004). Bioinformatics: Towards new directions for public health. *Methods of Information in Medicine, 43*(3), 208–214.

78 Maddox, T. M., Rumsfeld, J. S., & Payne, P. R. O. (2019). Questions for artificial intelligence in health care. *JAMA, 321*(1), 31–32.

79 Dhar, V. (2014). Big Data and predictive analytics in health care. *Big Data, 3*(2), 113–116.

80 Bourgois, P., Holmes, S. M., Sue, K., & Quesada, J. (2017). Structural vulnerability: Operationalizing the concept to address health disparities in clinical care. *Academic Medicine: Journal of the Association of American Medical Colleges, 92*(3), 299.

81 Rowe, J. P., & Lester, J. C. (2020). Artificial intelligence for personalized preventive adolescent healthcare. *Journal of Adolescent Health, 67*(2), S52–S58.

82 Merollini, K., Gordon, L. G., Aitken, J. F., & Kimlin, M. G. (2020). Lifetime costs of surviving cancer —A Queensland study (COS‐Q): Protocol of a large healthcare data linkage study. *International Journal of Environmental Research and Public Health, 17*(8), 2831.

83 Tham, J. (2010). Challenges to human dignity in the ecology movement. *The Linacre Quarterly, 77*(1), 53–62.

84 Taylor, D. R., & Lightbody, C. J. (2018). Futility and appropriateness: Challenging words, important concepts. *Postgraduate Medical Journal, 94*(1110), 238–243.

85 Kjetil, S., Hallet,J., Matthews, J. B., Schnitzbauer, A. A., Line, P. D., Lai, P. B. S., Otero, J., Callegaro, D., Warner, S. G., Baxter, N. N., Teh, C. S. C., Ng‐Kamstra, J., Meara, J. G., Hagander, L., & Lorenzon, L. (2020). Immediate and long‐term impact of the COVID‐19 pandemic on delivery of surgical services. *The British Journal of Surgery, 107*(10), 1250–1261. https://doi.org/10.1002/bjs.11670

86 Singh, P., Vijayan, R., Singh, E., & Mosahebi, A. (2019). Genetic editing in plastic surgery. *Aesthetic Surgery Journal, 39*(6), NP225–NP226.

87 Richie, C. (2018). A queer, feminist bioethics critique of facial feminization surgery. *The American Journal of Bioethics, 18*(12), 33–35.

88 Richie, C. (2019). Not sick: Liberal, trans, and crip feminist critiques of medicalization. *Journal of Bioethical Inquiry, 16*(3), 375–387.

89 Bernard, W. (1981). *Moral luck: Philosophical papers 1973‐1980*. Cambridge University Press.

90 Almeida, M., & Diogo, R. (2019). Human enhancement: Genetic engineering and evolution. *Evolution, Medicine, and Public Health, 2019*(1), 183–189.

91 Zhang, T., Xu, Y., Ren, J., Sun, L., & Liu, C. (2017). Inequality in the distribution of health resources and health services in China: Hospitals versus primary care institutions. *International Journal for Equity in Health, 16*(1), 42.

92 Shue, H. (1993). Subsistence emissions and luxury emissions. *Law & Policy, 15*(1), 39–60.

93 Beran, T. N., Ramirez‐Serrano, A., Vanderkooi, O. G., & Kuhn, S. (2015). Humanoid robotics in health care: An exploration of children's and parents' emotional reactions. *Journal of Health Psychology, 20*(7), 984–989.

94 Suwa, S., Tsujimura, M., Ide, H., Kodate, N., Ishimaru, M., Shimamura, A., & Yu, W. (2020). Home‐care professionals' ethical perceptions of the development and use of home‐care robots for older adults in Japan. *International Journal of Human–Computer Interaction, 36*(14), 1–9.

95 Dredge, S. (2015, February 27). Robear: The bear‐shaped nursing robot who'll look after you when you get old. *The Guardian*. https://www.theguardian.com/technology/2015/feb/27/robear-bear-shaped-nursing-care-robot

96 Cohen, I. G. (2020). Informed consent and medical artificial intelligence: What to tell the patient? *Georgetown Law Journal, 108*(6), 1425–1470.

97 Amankwah‐Amoah, J. (2017). Integrated vs. add‐on: A multidimensional conceptualisation of technology obsolescence. *Technological Forecasting and Social Change, 116*, 299–307.

98 Roberts, H., Cowls, J., Morley, J., Taddeo, M., Wang, V., & Floridi, L. (2020). The Chinese approach to artificial intelligence: An analysis of policy, ethics, and regulation. *AI & Society, 36*, 59–77. https://doi.org/10.1007/s00146-020-00992-2

99 Fuster, V. (2017). Changing demographics: A new approach to global health care due to the aging population. *Journal of the American College of Cardiology, 69*(24), 3002–3005.

100 Lancaster, K. (2019). The robotic touch: Why there is no good reason to prefer human nurses to carebots. *Philosophy in the Contemporary World, 25*(2), 88–109.

